# Patient-related factors, antibiotic prescribing and antimicrobial resistance of the commensal *Staphylococcus aureus* and *Streptococcus pneumoniae* in a healthy population - Hungarian results of the APRES study

**DOI:** 10.1186/s12879-019-3889-3

**Published:** 2019-03-13

**Authors:** László Róbert Kolozsvári, József Kónya, John Paget, Francois G. Schellevis, János Sándor, Gergő József Szőllősi, Szilvia Harsányi, Zoltán Jancsó, Imre Rurik

**Affiliations:** 1 Department of Family and Occupational Medicine, Faculty of Public Health, University of Debrecen, Debrecen, Hungary; 2 Department of Medical Microbiology, Faculty of Medicine, University of Debrecen, Debrecen, Hungary; 3 NIVEL, The Netherlands Institute for Health Services Research, Utrecht, The Netherlands; 4 Department of Preventive Medicine, Faculty of Public Health, University of Debrecen, Debrecen, Hungary; 5 Department of Health Systems Management, Faculty of Public Health, University of Debrecen, Debrecen, Hungary; 6 Department of General Practice & Elderly Care Medicine, Amsterdam Public Health Research Institute, VU University Medical Center, Amsterdam, the Netherlands

**Keywords:** Antimicrobial resistance, Antibacterial resistance, Commensal, Healthy population, Patient-related factors, *Staphylococcus aureus*, *Streptococcus pneumoniae*

## Abstract

**Background:**

Antimicrobial resistance (AMR) is an increasing public health problem worldwide.

We studied some patient-related factors that might influence the antimicrobial resistance.

and whether the volume of antibiotic prescribing of the primary care physicians correlate with the antibiotic resistance rates of commensal nasal *Staphylococcus aureus* and *Streptococcus pneumoniae*.

**Methods:**

The socio-demographic questionnaires, the antibiotic prescription and resistance data of commensal nasal *S. aureus* and *S. pneumoniae* were collected in the 20 participating Hungarian practices of the APRES study.

Multivariate logistic regression analyses were performed on the patient-related data and the antimicrobial resistance of the *S. aureus* and *S. pneumoniae* on individual, patient level.

Ecological analyses were performed with Spearman’s rank correlations at practice level, the analyses were performed in the whole sample (all practices) and in the cohorts of primary care practices taking care of adults (adult practices) or children (paediatric practices).

**Results:**

According to the multivariate model, age of the patients significantly influenced the antimicrobial resistance of the *S. aureus* (OR = 0.42, *p* = 0.004) and *S. pneumoniae* (OR = 0.89, *p* < 0.001). Living with children significantly increased the AMR of the *S. pneumoniae* (OR = 1.23, *p* = 0.019)*.* In the cohorts of adult or paediatric practices, neither the age nor other variables influenced the AMR of the *S. aureus* and *S. pneumoniae*.

At practice level, the prescribed volume of penicillins significantly correlated with the resistance rates of the *S. aureus* isolates to penicillin (rho = 0.57, *p* = 0.008). The volume of prescribed macrolides, lincosamides showed positive significant correlations with the *S. pneumoniae* resistance rates to clarithromycin and/or clindamycin in all practices (rho = 0.76, *p* = 0.001) and in the adult practices (rho = 0.63, *p* = 0.021).

**Conclusions:**

The age is an important influencing factor of antimicrobial resistance. The results also suggest that there may be an association between the antibiotic prescribing of the primary care providers and the antibiotic resistance of the commensal *S. aureus* and *S. pneumoniae*. The role of the primary care physicians in the appropriate antibiotic prescribing is very important to avoid the antibiotic resistance.

## Background

Antimicrobial resistance (AMR), in particular antibacterial resistance (ABR) is an increasing public health problem worldwide [1]. The available antibiotics might become ineffective against infections due to antimicrobial resistance, although there is a continuous research and development of new antibiotics. There are only a limited number of new antibiotics available in primary care [2], so avoiding resistance with all the possible means is very important.

About 90% of the antibiotics are prescribed by primary care providers [3, 4].

It has been shown that the overuse of antibiotics increases the risk of side effects, raises costs and is associated with increased AMR levels [5]. The resistance rates of the bacteria are influenced by prescribers’ prescribing habits and by health policies as well [6].

Higher prescribing rates of antibiotics may increase the resistance in individuals, on the community level and in the whole society [7–9]. The results of the analysis of the outpatient antibiotic use and resistance in 26 countries in Europe showed significant correlation between increased consumption of antibiotics and the resistance of the microorganisms at national level [3].

Antibiotic use is a major contributing factor to the development of resistant bacteria, but many other factors may contribute: e.g. patients’ age, movement and migration, socio-economic factors, dose and duration of the antibiotic treatment, gene transfer and clonal spread [10]. Human beings carry a very large number of commensal organisms which probably provide a significant but underestimated reservoir of resistance genes [11]. How these influence the development of resistance in pathogenic organisms is as yet uncertain [12].

The APRES study (The Appropriateness of PREScribing antibiotics in primary health care in Europe with respect to antibiotic resistance) was designed to establish resistance patterns of *Staphylococcus aureus* and *Streptococcus pneumoniae* in the nasal flora in community-dwelling individuals and to collect data about the antibiotic prescribing patterns of primary care providers (family physicians/general practitioners, primary care paediatricians) in nine European countries, the project was closed in 2014 [9, 13, 14].

The results of the APRES study in other countries suggest that age is one of the major determining factors of the prevalence of *S. aureus* carriership, but there may be also other factors that influence the nasal carriage [15, 16].

Another previously published result of the APRES study suggests that higher resistance rates of *S. aureus* are associated with younger age, but also the primary care physicians’ antibiotic prescribing behaviour may influence the odds of the carriage of the resistant nasal commensal *S. aureus* and there is a need for further investigations on a national level [17].

The antibiotics are prescription only medicines, that can only be prescribed by doctors in Hungary. The national total antibiotic consumption was relatively constant between 1996 and 2003, but there were large differences between the community antibiotic consumption of the regions and between counties [18, 19]. Other previously published Hungarian studies about the ambulatory antibiotic consumption established a constant quantity (18.0 ± 1.8 DDD/1000 inhabitants/day) between the years 2006–2010 [20].

Primary care practices are usually the first point of access to health care in Hungary. There are about 6500 primary care (PC) practices for a population of approximately 10 million in Hungary. There are three main types of PC practices in Hungary: *adult practices* (n ≈ 3500) treating adults only, *paediatric practices* (n ≈ 1500) with primary care paediatricians mostly in bigger towns and cities, caring of children under 18 years only, and *mixed practices* (n ≈ 1500) treating people of all ages (mainly established in smaller settlements, villages) [21].

The antimicrobial resistance at younger patients may be higher and the primary care providers antibiotic prescribing may influence the resistance rates of the commensal *S. aureus* and *S. pneumoniae*, we found no previous publications that investigated the antimicrobial resistance of two commensal bacteria at individual and at primary care practice level*.*

The aim of this study was to investigate, based on the Hungarian data of the APRES Study:

1. The patient-related factors that may influence the antimicrobial resistance of the commensal *S. aureus* and *S. pneumoniae* at patient level*.*
2. Whether the volume of antibiotic prescribing of primary care providers correlate with the antibiotic resistance rates at practice level.

## Methods

The APRES study design was published in previous articles in detail [13, 14], we therefore mainly focus on the methodology related to our study.

### Patients and practices

Socio-demographic data of the participating patients (eg. age, gender, living with children, number of GP visits during the previous year etc.) and data of the practices (e.g. number of registered patients, practice type etc.) were collected with questionnaires.

The Hungarian part of the study was conducted in 20 primary care practices, patients were selected in all 3 types of Hungarian primary care practices (13 adult, 5 paediatric and 2 mixed practices) between November 2010 and March 2011.

The number of participating patients and primary care practices were calculated and established by an international group of experts and included in the APRES Study protocol [13, 14].

The participating practices were located in 5 counties (Borsod-Abaúj-Zemplén, Csongrád, Hajdú-Bihar, Heves, Szabolcs-Szatmár-Bereg) and in the capital (Budapest). The practice locations represented the main types of settlements across Hungary: the capital (1), larger cities (10), towns (5), large village (1) and villages (3 practices).

### Bacterial specimens

Bacterial specimens were collected from a random sample of persons over 4 years of age, who attended the primary care practices and had no symptoms of an infectious disease. Other exclusion criteria were antibiotic use or hospitalisation in past 3 months, severe health problems (terminal illness, immunocompromised).

Nasal swabs were obtained from the individuals according to a routine protocol. The specimens were immediately inserted into a container tube, with charcoal transport medium and transferred into the Department of Medical Microbiology at the University of Debrecen within 48 h. Colonies with characteristic Staphylococcus morphology were identified as *S. aureus* based on positive coagulase test and colonies of *S. pneumoniae* were identified on the basis of colony morphology and resistance to optochin by the national laboratory, then transferred and tested for resistance against antibiotics in one central laboratory (Maastricht University). Both *S. aureus* and *S. pneumoniae* isolates were stored in frozen skimmed milk under liquid nitrogen until transporting them to Maastricht University where antibiotic resistance were established quantitatively with a broth dilution method in micro-titre plates according to EUCAST recommendations [13, 22]. The *S. aureus* and *S. pneumoniae* isolates were tested for the following antimicrobials: penicillins with and without beta-lactamase inhibitors, cephalosporines, macrolides, tetracyclines, quinolones, trimethoprim-sulfamethoxazole and rifampicin; for *S. aureus* also aminoglycosides, fusidic acid and mupirocin were tested [13, 14].

Resistance was considered present if there was resistance to one or more antibiotic (R ≥ 1). Furthermore, we considered the resistance rates to penicillin (J01C) and azithromycin and/or clindamycin (J01F) specifically. The resistance rates were also calculated in cohorts of practices (all practices, adult practices, paediatric practice).

### Antibiotic prescribing

The antibiotic prescribing data were collected from the 20 primary care physicians’ computer databases. All individual antibiotic prescriptions were classified by the active substance according to anatomic therapeutic classification (ATC), DDD (Defined Daily Dose), DPP (dose per package) and number of packages. In our study, the antibiotic prescribing of the primary care practices was expressed in DDD/1000 active patients/day, according to the WHO ATC/DDD methodology [23]. The antibiotic exposure was measured with the DDDs (not the number of packages), because this may be more appropriate for international and national comparison and to evaluate the correlation between the use of antibiotics and antimicrobial resistance [4, 24, 25]. There are opinions, that the main effect of the prescribed antibiotics to the resistance rates may last for up to one year, so we considered the prescribing data the year preceding the nasal sampling, in 2010 [8, 17, 26].

### Statistical analysis

Multivariate logistic regression models were performed on the patient-related data and the antimicrobial resistance (isolates that were resistant to at least one antibiotics, R ≥ 1) of the *S. aureus* and *S. pneumoniae* on individual, patient level. The association between the *S. aureus* and *S. pneumoniae* antimicrobial resistance (outcome/dependent variables) and the patient- related factors (explanatory/independent variables) were analysed.

The patient-related factors (independent variables) were patients’ age (years), gender (female/male), job (working with livestock and/or in nursery and/or in healthcare), living with children (yes/no), number of GP visits during the previous year (1–4 visits/none, 5 + visits/none). The analyses were performed in the whole sample (all practices) and in the cohorts of practices taking care of adult persons (adult practices) or children only (paediatric practices).

Due to the data collection methods and anonymous patient data, the antimicrobial resistance and individual antibiotic prescribing data could not be linked at individual level, this was one the reasons why we investigated the correlation between the antibiotic prescribing and resistance rates at practice level. The resistance rates were also calculated at practice level. We presumed that the most commonly prescribed antibiotics groups in primary care are the penicillins (*Beta-lactam antibacterials, penicillins J01C*) and macrolides (*Macrolides, lincosamides and streptogramins* J01F). Furthermore, we considered the resistance rates to penicillin (J01C) and azithromycin/clarithromycin and/or clindamycin (J01F) specifically. The associations between the usage of antibiotics (DDD/1000 patients/day) and the resistance rates were analysed by Spearman’s rank correlation. The correlations were analysed in the whole sample (all practices) and we also calculated the correlation in the cohorts of adult practices and paediatric practices.

The statistical analyses were performed with STATA 11.1 software (Statacorp LP. College Station, TX, USA).

## Results

### Patients and practices

There were 4017 questionnaires collected from the participating patients. The age distribution of the participants was 4–19 years: 28.2%, 20–64 years: 50.4%, and above 65 years: 21.3%. Their gender distribution was 44.5% male (*n* = 1713), 55.5% female (*n* = 2137).

The average number of *registered patients* was 1905 persons per practice (range between 724 and 3308). The adult practices had 2219 registered patients on average, the mixed practices 1719 and the paediatric practices 1163 patients on their list in year 2010, respectively.

The number of *active patients* (patients who attended the primary care surgery at least once in 2010) was used as denominator in our ecological statistical analyses. The average number of *active patients* was 1534 persons per practice (range between 658 and 1976). On average, there were 1758 active patients in the adult, 1227 in the mixed practices and 1057 in the paediatric practices seen in year 2010, respectively.

### Bacterial specimens

There were 4017 nasal swabs collected, the number of the incorrect sampling was 150 (absence of bacteria, including normal microbiota), the number of mismatched samples were 17 (absence of patient background information or laboratory data). 3850 swabs were tested in the laboratory, including 2471 swabs from the adult and 984 in the paediatric practices.

The prevalence of carrying commensal *S. aureus* was 14.1% in all practices (*n* = 541), 12.8% in adult practices (*n* = 317) and in 17.6% paediatric practices (*n* = 173). The prevalence of the *S. pneumoniae* was 4.1% in all practices (*n* = 159), 1.1% in adult practices (*n* = 28) and 12.1% in paediatric practices (*n* = 119).

The number and percentage of resistant isolates are shown in Table 1.

**Table 1:** The resistance of *S. aureus* and *S. pneumoniae* in all practices, adult practices and paediatric practices

Resistance	All practices	Adult practices	Paediatric practices
*S. aureus*	*n* = 541	*n* = 317	*n* = 173
R ≥ 1	434 (80.2%)	237 (74.8%)	151 (87.3%)
J01C	410 (75.8%)	221 (69.7%)	145 (83.8%)
J01F	65 (12.0%)	32 (10.1%)	28 (16.2%)
*S. pneumoniae*	*n* = 159	*n* = 28	*n* = 119
R ≥ 1	112 (70.4%)	10 (35.7%)	95 (79.8%)
J01C	30 (18.9%)	3 (10.7%)	25 (21.0%)
J01F	18 (11.3%)	2 (7.1%)	15 (12.6%)

[R ≥ 1, resistance to one or more antibiotics; J01C resistance to penicillins; J01F resistance to macrolides/lincosamides]

According to our descriptive statistics, the prevalence of antimicrobial resistance (resistance to one or more antibiotics (R ≥ 1) and resistance against penicillins (J01C) and macrolides/lincosamides (J01F) of the *S. aureus* and *S. pneumoniae* seemed to be lower in the cohorts of adult practices and higher in the paediatric practices, comparing to the cohorts of all practices.

### Antibiotic prescribing

There were 24,223 antibiotic prescriptions issued by the participating primary care providers in 2010. The average volume of all prescribed antibiotics was 15.86 DDD/1000 active patients/day per practice, the most commonly prescribed antibiotics were the *beta-lactam antibacterials, penicillins* (ATC3 level J01C; 8.61 DDD/1000 active patients/day per practice), the *macrolides/lincosamides* (J01F; 3.47), the *other beta-lactam antibacterials* (J01D; 1.37); the *quinolone antibacterials* (J01 M; 1.17), the *tetracyclines* (J01A; 0.51), the *sulphonamides and trimethoprim* (J01E; 0.42), and the *other antibacterials* (J01X; 0.98).

The mean volume of the prescribed penicillins (J01C) was 8.86 ± 1.8 (mean ± SD) DDD per 1000 active patients/day in all practices, 4.96 ± 2.74 DDD per 1000 active patients/day in the adult practices. The mean volume of the prescribed macrolides/lincosamides (J01F) was 3.43 ± 2.39 DDD per 1000 active patients/day in all practices, 2.79 ± 1.17 DDD per 1000 active patients/day in the adult practices.

#### Patient-related factors and antimicrobial resistance of the *S. aureus* and *S. pneumoniae*

The age of the patients significantly influenced the resistance of the *S. aureus* and *S. pneumoniae.* The odds having antimicrobial resistance decreased in the group of the patients over 18 years of age (OR_S.aureus_ = 0.42, *p* = 0.004; OR_S.pneumoniae_ = 0.89, *p* < 0.001). Living with children significantly increased (OR = 1.23, *p* = 0.019) the AMR of the *S. pneumoniae*.

It appears that in the cohorts of adult and paediatric practices the age-related or other variables did not influence the AMR of *S. aureus* and *S. pneumoniae* (Table 2)*.*

**Table 2:** Multivariate logistic regression models on the patient-related variables and antimicrobial resistance of the *S. aureus* and *S. pneumoniae*

Variables	*S. aureus* resistance					
	All practices		Adult practices		Paediatric practices	
	Odds Ratio	*p*-value	Odds Ratio	*p*-value	Odds Ratio	*p*-value
Patient variables						
Age groups (over 18 years/under 18 years)	**0.42**	**0.004**	0.64	0.693	0.44	0.269
Gender (male/female)	0.79	0.322	0.92	0.765	0.42	0.083
Job - Livestock (yes/no)	0.91	0.970	1.01	0.980	N/A	N/A
Job - Nursery (yes/no)	0.55	0.329	0.89	0.967	N/A	N/A
Job - Healthcare (yes/no)	0.90	0.903	0.95	0.952	N/A	N/A
Living with children (yes/no)	0.79	0.537	0.67	0.428	1.12	0.859
Number of GP visits						
1–4 visits/none	0.60	0.528	0.77	0.757	0.11	0.969
5 + visits/none	0.90	0.898	1.18	0.859	0.10	0.971
Variables	*S. pneumoniae* resistance					
	All practices		Adult practices		Paediatric practices	
	Odds Ratio	*p*-value	Odds Ratio	*p*-value	Odds Ratio	*p*-value
Patient variables						
Age groups (over 18 years/under 18 years)	**0.89**	**< 0.001**	1.00	0.842	0.79	0.785
Gender (male/female)	1.45	0.425	1.87	0.503	1.27	0.646
Job - Livestock (yes/no)	1.23	0.873	3.59	0.430	N/A	N/A
Job - Nursery (yes/no)	N/A	N/A	N/A	N/A	N/A	N/A
Job - Healthcare (yes/no)	3.71	0.324	3.07	0.206	N/A	N/A
Living with children (yes/no)	**1.23**	**0.019**	0.29	0.409	0.34	0.085
Number of GP visits						
1–4 visits/none	0.98	0.988	1.37	0.962	1.08	0.933
5 + visits/none	0.98	0.925	0.93	0.409	1.03	0.978

[significant results marked in bold]

#### The correlation between antibiotic prescribing and resistance rates

The volume of prescribed penicillins (J01C) correlated significantly with the resistance rates of *S. aureus* isolates to penicillin, when including the data from all practices in the analyses (rho = 0.57, *p* = 0.008), this means a moderate positive correlation, but not correlated when considering the adult (rho = 0.30, *p* = 0.319) or paediatric (rho = 0.54, *p* = 0.284) practices only. There was no significant correlation between the volume of prescribed macrolides, lincosamides (J01F) and the *S. aureus* resistance rates to azithromycin and/or clindamycin (Table 3).

**Table 3:** Correlations between volume of prescribed penicillins and macrolides/lincosamides and resistance rates of *S. aureus* (%) in all practices, adult and paediatric practices

**All practices**			
Prescription of	***S. aureus*** resistance to	rho	*p*-value
**Penicillins (J01C)**	**Penicillin**	**0.57**	**0.008**
Macrolides, lincosamides (J01F)	Azithromycin and/or clindamycin	0.22	0.343
**Adult practices**			
Prescription of	***S. aureus*** resistance to	rho	*p*-value
Penicillins (J01C)	Penicillin	0.30	0.319
Macrolides, lincosamides (J01F)	Azithromycin and/or clindamycin	0.03	0.922
**Paediatric practices**			
Prescription of	***S. aureus*** resistance to	rho	*p*-value
Penicillins (J01C)	Penicillin	0.60	0.284
Macrolides, lincosamides (J01F)	Azithromycin and/or clindamycin	0.54	0.391

(Spearman’s rank correlation) [significant results marked in bold]

The volume of prescribed macrolides, lincosamides (J01F) showed strong positive significant correlation with the *S. pneumoniae* resistance rates to clarithromycin and/or clindamycin in all practices (rho = 0.76, *p* < 0.001) and in the adult practices (rho = 0.63, *p* = 0.021), but no correlation in the paediatric practices (rho = 0.52, *p* = 0.191). The correlation between the volume of prescribed penicillins (J01C) with the resistance rates of *S. pneumoniae* was not statistically significant (Table 4.)

**Table 4:** Correlations between volume of prescribed penicillins and clarithromycin and/or clindamycin and resistance rates (%) in all practices, adult and paediatric practices

**All practices**			
Prescription of	***S. pneumoniae*** resistance to	rho	*p*-value
Penicillins (J01C)	Penicillin	0.39	0.084
**Macrolides, lincosamides (J01F)**	**Clarithromycin and/or clindamycin**	**0.76**	**0.001**
**Adult practices**			
Prescription of	***S. pneumoniae*** resistance to	rho	p-value
Penicillins (J01C)	Penicillin	0.30	0.319
**Macrolides, lincosamides (J01F)**	**Clarithromycin and/or clindamycin**	**0.63**	**0.021**
**Paediatric practices**			
Prescription of	***S. pneumoniae*** resistance to	rho	p-value
Penicillins (J01C)	Penicillin	0.10	0.872
Macrolides, lincosamides (J01F)	Clarithromycin and/or clindamycin	0.52	0.191

(Spearman’s rank correlation) [significant results marked in bold]

## Discussion

The age of the patients may be an important factor regarding antimicrobial resistance. However, when we investigated the cohorts of both adult and paediatric practices separately, there was no association between the age and resistance. Living with children in a same household is likely to increase the risk of having resistant *S. pneumoniae* isolates. Childhood seems to be a more important influencing factor than the age only, as it could be concluded from the previous publications [14, 16, 17]. The APRES Study, that studied apparently healthy population revealed reduced prevalence of both methicillin-sensitive and methicillin-resistant *S. aureus* isolates with older age, but the prevalence and resistance differed between and within the participating countries [14, 16, 17]. Methicillin-resistant *S. aureus* isolates from patients with impaired health showed increasing fluoroquinolone resistance rate with patients’ age. The latter phenomenon was however unique for fluoroquinolones only and was not noticed for other antibiotics [27]. In the healthy population, the resistance rates of different age groups with recent history of increased antibiotic load are likely different from the patients with impaired health [14, 27].

The prescribed average volume of all prescribed antibiotics by the participating Hungarian physicians was 15.86 DDD/1000 active patients/day per practice in 2010. The most commonly prescribed antibiotics were the *beta-lactam antibacterials, penicillins* and the *macrolides*. According to the data from the European Surveillance of Antimicrobial Consumption (ESAC) project the consumption of antibacterials for systemic use (ATC group J01) was 15.67 DDD/1000 inhabitants/day in the primary care sector in Hungary in 2010, this is comparable to the prescribed average volume of the antibiotics in the cohort of the 20 practices in our study [4].

The nasal carriage of the commensal *S. aureus* and *S. pneumoniae* were higher in the paediatric practices. There were differences between the antimicrobial resistance (resistance to one or more antibiotics - R ≥ 1) of *S. aureus* and *S. pneumoniae* in the participating primary care practices, the lowest resistance rates were found in the cohort of the adult practices.

There may be an association between the volume of prescribed antibiotics by primary care physicians and the bacterial resistance rates, the results of the Spearman’s rank correlation analysis suggested, that the primary care providers’ antibiotic prescribing may correlate with the antibiotic resistance of the commensal *S. aureus* and *S. pneumoniae*.

### Strengths and limitations

There was large amount of information collected. The average volume of the antibiotic prescribing by the participating practices in our study was similar to the previously published Hungarian outpatient antibiotic prescribing data, so the volume of antibiotic prescribing in the participating practices was likely representative for the Hungarian practices [4, 19, 20]. Other strength of the study was the large sample size of the questionnaires and nasal swabs taken from a healthy population. The prevalence and resistance rates were similar to previously published data, but most studies investigated the resistance rates from the hospital settings or from pathogen bacteria [3, 8, 12]. There was no previous study found which investigated the correlation between the antibiotic prescribing of the primary care providers at practice level and the antibiotic resistance of the commensal flora. Previous publication from the APRES Study suggested, that AMR could be associated with the antibiotic exposure in the primary care, it is important to investigate the correlation between the GP practices antibiotic prescribing and the prevalence and resistance of the commensal bacteria on a national level, as we done in our publication [17].

A limitation of the study was the relatively small number of the participating patients and practices, comparing to the total number of primary care practices and to the total population of Hungary, even though the expected number of the practices and participants per country were analysed, calculated and established by an international group of experts and included in the study protocol. The sample was selected as a likely representative sample to the Hungarian primary care practices [13]. The number of the incorrect sampling and the mismatched samples caused some loss of the prevalence and resistance data. The low prevalence and antibacterial resistance of the commensal *S. pneumoniae* made the statistical analyses less powerful. The correlations in our study does not necessarily imply causality.

The antimicrobial resistance can be affected by several factors, some of them cannot be influenced (eg. age of the patient), but there are factors, like the primary care providers’ antibiotic prescribing, that can be influenced by education, regulation of the prescribing, developing and use of guidelines etc.

Antibiotic prescribing in the primary care practices may influence the resistance of the commensal flora, which could be a reservoir of resistance genes. According to previous studies, this resistance likely can be transferred from the commensal flora to the pathogens [10, 11]. Primary care providers prescribe large proportions of the antibiotics; therefore, they have an important role to prevent the spread of the antibiotic resistance. It is very important that the healthcare providers only should prescribe antibiotics only when it’s necessary and when the antibiotic treatment is indicated. This can be achieved with appropriate health policies, guidelines and continuous education of the primary care physicians.

## Conclusions

The age is an important influencing factor of antimicrobial resistance. The results suggest that there may be an association between the antibiotic prescribing of the primary care providers and the antibiotic resistance of the commensal *S. aureus* and *S. pneumoniae*. The role of the primary care physicians in the appropriate prescribing is very important to avoid the antimicrobial resistance.

## Abbreviations

ABR: Antibacterial resistance
AMR: Antimicrobial resistance
APRES: The Appropriateness of PREScribing antibiotics in primary health care in Europe with respect to antibiotic resistance
DDD: Defined Daily Dose
DPP: Dose per package
ESAC: European Surveillance of Antimicrobial Consumption
EUCAST: The European Committee on Antimicrobial Susceptibility Testing
GP: General Practitioner
MRSA: Methicillin-resistant *Staphylococcus aureus*
PC: Primary Care
WHO: World Health Organization
